# Analysis of the potential application of a residential composite energy storage system based on a double-layer optimization model

**DOI:** 10.1038/s41598-024-56414-6

**Published:** 2024-03-15

**Authors:** Xueyuan Zhao, Xiaoyu Ying, Weijun Gao, Fanyue Qian, Yang Tan, Jing Xie

**Affiliations:** 1 Institute of Architectural Engineering, Zhejiang University, Hangzhou, 310058 China; 2 School of Spatial Planning and Design, Hangzhou City University, Hangzhou, 310015 China; 3 Faculty of Environmental Engineering, The University of Kitakyushu, Kitakyushu, 808-0135 Japan; 4 Institute of Mechanical Engineering, Tongji University, Siping Road, Shanghai, 1239 China

**Keywords:** Composite energy system, User-side energy storage, Comprehensive performance comparison, Double-level optimization, Adaptive particle swarm optimization algorithm, Energy storage, Renewable energy

## Abstract

Along with the further integration of demand management and renewable energy technology, making optimal use of energy storage devices and coordinating operation with other devices are key. The present study takes into account the current situation of power storage equipment. Based on one year of measured data, four cases are designed for a composite energy storage system (ESS). In this paper, a two-tiered optimization model is proposed and is used to optimizing the capacity of power storage devices and the yearly production of the system. Furthermore, this paper performs a comparative analysis of the performance of the four cases from the energy, environmental and economic perspectives. It is concluded that this kind of energy storage equipment can enhance the economics and environment of residential energy systems. The thermal energy storage system (TESS) has the shortest payback period (7.84 years), and the CO_2_ emissions are the lowest. Coupled with future price volatility and the carbon tax, the electrothermal hybrid energy storage system (HESS) has good development potential. However, the current investment cost is very high, and it will not be possible to recover this cost in 10 years. Finally, it is recommended that the cost of equipment be reduced in combination with subsidies and incentives for further promotion. The research results not only fill a gap in the study area, but also provide some suggestions for further development of industry and research on user-side energy storage.

## Introduction

Electricity is a crucial foundation for energy supply and the sustainable development of society and the economy. However, sustained increases in energy requirements and reductions in primary energy consumption pose challenges for sustainable development. To solve this problem, distributed energy resources, mainly renewable, have gained recognition are being actively development worldwide, and are becoming a crucial topic in new energy research^1^. Distributed generation technology based on renewable energy is an important supplement to traditional power generation technology^2^. As urbanization accelerates, total electricity consumption continues to increase, especially household electricity consumption, which increases the electricity consumption of society as a whole^3,4^. Consequently, the housing allocated energy system has received extensive attention as a concept and method of flexible energy saving. However, with many distributed power sources and extensive research on the network, the instability and loss of control of network power have gradually emerged^5,6^.

To ameliorate these adverse effects, we should take full account of the economical and environment benefits of distribution grids, and enhance the utilization efficiency of renewable energy systems(RESs). Interested researchers need to explore a more sophisticated approach to networking. A number of countries have developed their own strategies, integrated micro network architecture, and set up relevant laboratories and demonstration programs. Research and demonstrations of community and insular microgrid systems have been conducted in a number of countries^7,8^. However, the disadvantages of microgrids in load matching and technology commercialization still exist^9,10^. Therefore, energy optimization management techniques such as flexible resource scheduling for direct current(DC) power supply demand side response are particularly important^11,12^. In particular, energy storage technology that can quickly balance the power fluctuations of microgrids, thus guaranteeing the security and reliability of power supply and the efficiency of energy distribution^13,14^, can effectively highlight the advantages of microgrids in terms of reliability and flexibility^15^.

The characteristics of energy storage systems (ESSs), which have a wide application range, flexible dispatch ability and high grid friendliness, compensate for the shortage of microgrid technology, and have a positive impact on the application and promotion of ESSs^16^. However, the high initial investment and long payback periods (PPs) are significant challenges that limit the economic development of these systems. Therefore, the optimization of ESS design and management is an essential issue for further research^17^. Several researchers have made notable contributions to this field. Nero performed a high-quality comparative analysis of a DC control system consisting of a public alternating current(AC) network, a battery storage system (BESS), distributed generators, and user loads^18^. Li was able to optimize the mix size of photovoltaic(PV) and battery home systems based on the genetic algorithm(GA) under time of use (TOU) to meet load demands and save 2457.8 USD in electricity costs per year^19^. Anathema designed a distributed control scheme that combines household batteries and a PV element to solve the overvoltage problem due to the high transmission rate of PV elements^20^. Li simulated and analyzed a hybrid heat pump and thermal energy storage system(TESS). Compared with traditional nonthermal storage systems, this approach reduces the annual grid power demand by approximately 76%^21^.

While the promotion of residential ESSs is economically restricted, the lack of an optimization strategy and evaluation method for the final choice of operation system is also one of the obstacles in the promotion process^22^. Remus evaluated the effects of weather and working patterns on residual load, and suggested that both factors should be considered when selecting the optimum battery size^23^. Astor analyzed the economic viability of BESSs by combining various economic indicators, such as energy tariffs, the rate of return, net costs, energy leveling costs, and CO_2_ reductions^24^. The results confirmed that BESS investment in housing is profitable. Using the example of Australian family housing, Mulleriyawage claimed that, given sufficient subsidies, installing a BESS based on current market prices can be economically profitable^25^. Romani used an optimization algorithm to simulate and test the results of BESS charging and discharging, with the goal of limiting peak pressure and flattening the power curve^26^.

At present, some studies have analyzed and summarized the application of energy storage for smoothing energy output fluctuations, assisting grid connections, participating in frequency modulation and alleviating peak shaving pressure. A residential ESS converts consumers from users of community- and island-level microgrid systems to participants in energy optimization management. However, due to differences in service objects, configuration environments, business models and other influencing factors, there will be obvious differences in ESSs. In the application of residential energy storage, the profit return from the promotion of energy storage is an important factor affecting the motivation of users to install energy storage. Therefore, under the price policy and market environment, the application scenario selection and benefit analysis of user-side energy storage are particularly important. Currently, the application and optimization of residential energy storage have focused mostly on batteries, with little consideration given to other forms of energy storage. Based on the load characteristics of users, this paper proposes a composite energy system that applies solar, electric, thermal and other types of energy. It studies the application potential of residential energy storage, and it designs four cases in different scenarios. It optimizes the size and output of energy storage equipment in the cases and compares system performance under the different scenarios. The aim is to reasonably match the supply and storage equipment in the residential energy system and to use user-side energy storage to achieve peak shaving, energy conservation and emission reduction.

The rest of the paper is organized as follows: Section .“Research objective and system composition” presents the structure of the home energy management system(HEMS) and devices for storing energy, basic information, and energy consumption data on the research object. Section “Model and research method” develops a double-layer optimization model and comprehensive comparison model, and describes research methods. Then, the optimum dimensions of the energy storage device are determined based on the optimum result for the top level, and energy consumption under different scenarios is simulated based on the optimal size to complete the lower layer optimization in Section “Results and discussion”. Based on the results obtained, the cases are compared from the three perspectives of energy, economics and the environment. Meanwhile, the sensitivity of the case is analyzed in combination with the policy and price fluctuations. Finally, conclusions are drawn in Section “Conclusions”.

## Research objective and system composition

### Research objective and basic data

Following the "Great East Japan Earthquake", Japan shut down a large number of nuclear power stations, which caused a peak in hourly electricity distribution. The Japanese government subsequently paid greater attention to investing in the energy framework and began to rethink its strategy for intelligent grids^27^. Japan provides three levels of building infrastructure, namely, the country, regional, and household levels, considering different grid models. At the household level, there are intelligent houses, zero-energy homes, batteries, electric vehicles (EVs), etc., which focus on energy efficiency and CO_2_ emissions^28^. HEMSs are widely applied in intelligent homes as a two-way communication center to control energy consumption and the transfer of information. The HEMS implements unified management and centralized control of distributed generation, power consumption and energy storage equipment to optimize energy consumption.

**Figure 1:**
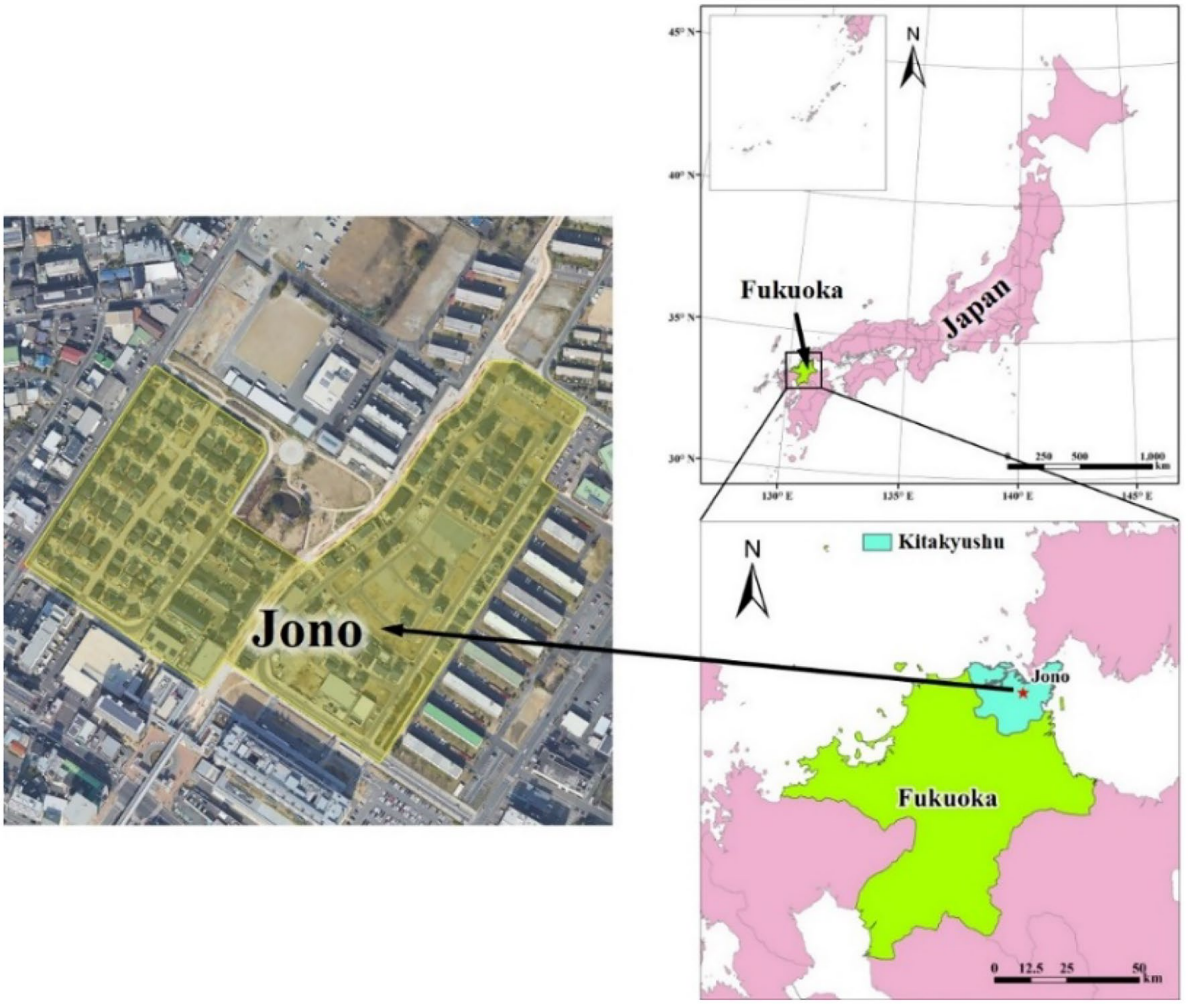
Location of the Jono Zero Carbon Advanced Urban Area. (Generated by Arc GIS10.8, http://3.sylqkji.cn/arcgis/).

**Figure 2:**
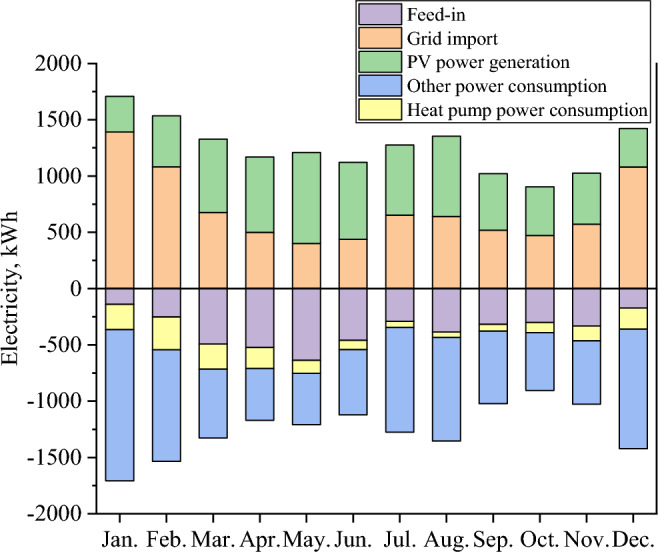
Monthly energy consumption of the target residence.

**Figure 3:**
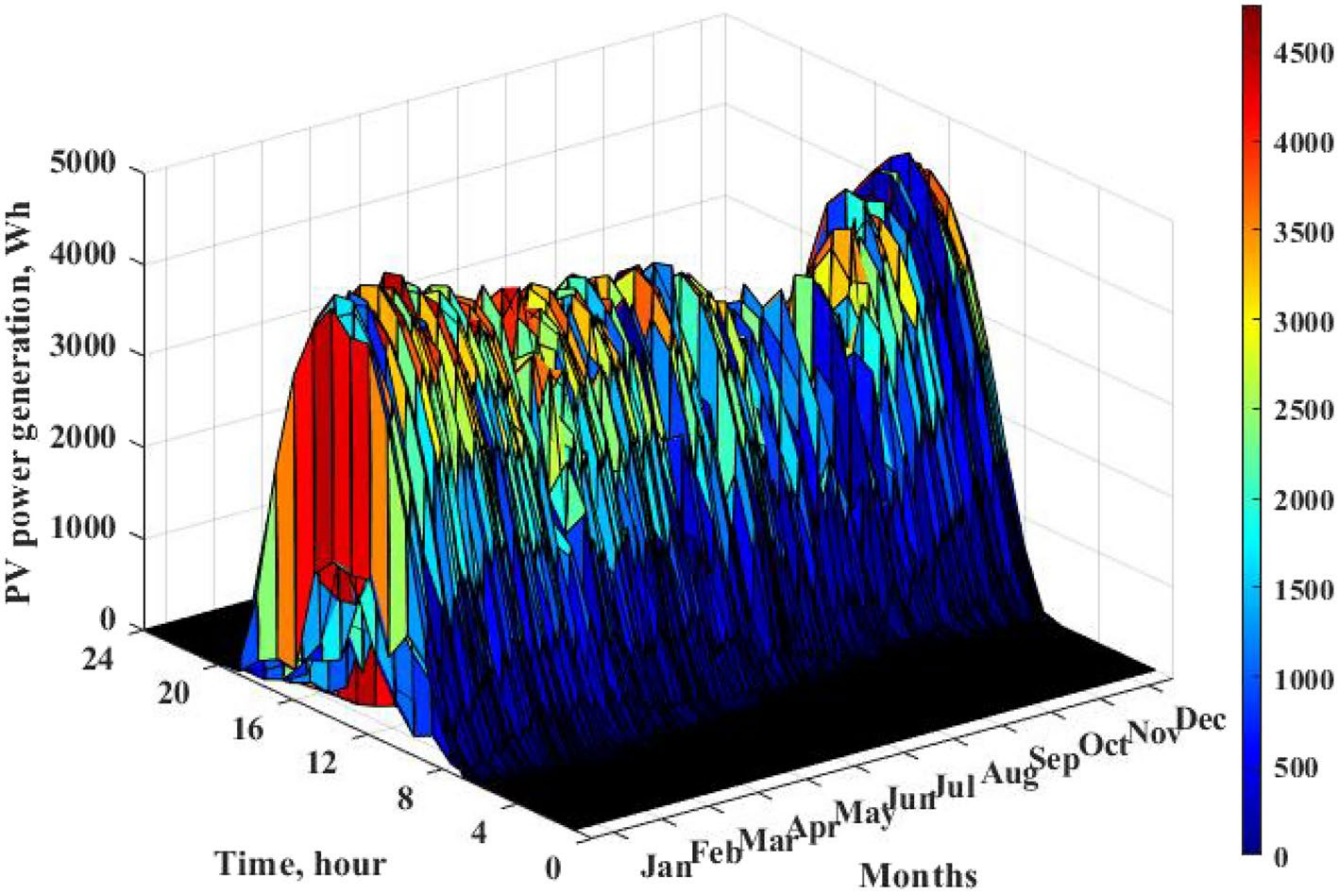
Monthly PV power generation of the target residence.

Since 2010, to alleviate pressure on the power grid and reduce CO_2_ emissions, the Ministry of Economy, Trade and Industry (METI) of Japan has carried out five-year smart grid demonstration projects in Yokohama, Toyota, Keisanai, and Kitakyushu. The Jono Zero Carbon Advanced Urban Area is one of the major projects of Kitakyushu’s environmental future city initiative, and it is striving for a theoretical 100% reduction in carbon emissions across the entire district. To achieve this goal, the city entered into an agreement with the developer that requires energy management measures to establish an energy management system in each home, with part of the installation cost assumed by the city. In this study, we selected houses located in developed cities with zero carbon emissions. Figure 1 shows the location of the Jono Zero Carbon Advanced Urban Area in Japan. The home energy system of households adopted an all-electric system combining PVs and a heat pump. This paper monitored electricity consumption every hour of every day from April 1, 2017, to March 31, 2018. The basic data on the target residence are given in Table 1, and the energy consumption per month is shown in Fig. 2. The energy consumption system of the study residence is an all-electric system, and according to the energy conservation rules, energy consumption is divided into five parts. As the heating equipment of the energy system, the heat pump consumes electricity to meet the thermal demand of users. Due to weather and temperature, the heat pump consumes more electricity in winter and less in summer. Therefore, in the monthly energy consumption comparison, over the course of one year, the power consumption in winter is generally greater than that in summer. The energy supply part is composed of grid imports and PV power generation. The generation of photoelectric energy, which is the major energy source in the system, is displayed in Fig. 3. The working hours of PVs are concentrated from 6:00 to 18:00, and the electricity generation in summer is significantly higher than that in winter. According to the feed-in tariff (FiT) policy, the feed-in component functions when PV generation meets the electricity demand of users and there is residual power, which can be fed back to the public grid.

**Table 1:** Basic information of the target residence.

Residential type	Detached two-story building
Total area	120 m^2^
Range of measurement data	2017-04-01–2018-03-31
Residential energy system	PV + heat pump
Number of family members	4

### ESS composition and study case

**Figure 4:**
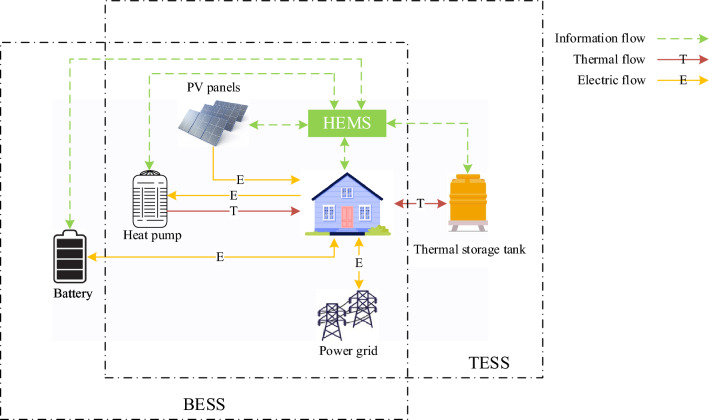
Structure of residential energy system with energy storage equipment.

As a support scheme for PV technology, the FiT policy has contributed to the development and wide use of optoelectronics. In the early stage of residential PV system promotion, due to the high equipment price, the government proposed a series of encouraging policies, including subsidies for installation costs and the FiT^29^. However, as the price of the system has dropped and as solar power generation becomes increasingly popular, Japan's government has begun to reduce its FiT^30^. Doing so has cast doubt on the economic feasibility of solar power generation systems. Not only does our proposed system address these issues, but it also assists the system in tackling the issues associated with intermittent PV generation^31^. The system architecture is composed of power stream and data stream, which can be exchanged between the HEMS client and the power company in real-time. The primary objective is to visualize energy by means of automatic bidirectional data transmission and optimum management of home appliances. Figure 4 shows the equipment composition and energy flow structure of the residential energy system in this study. PVs and batteries are the main power supply equipment, while heat pumps and heat storage tanks are the main heating equipment. At the same time, the system introduces batteries and heat storage tanks as storage devices for electrical and thermal energy, respectively. Surplus solar energy can also be returned to the public network, and if the power of the network cannot satisfy the energy demand of users, the shortage can be filled by purchasing a shared network.Four cases were generated in accordance with different circumstances, as shown in Table 2.

**Table 2:** Case introduction.

Case	Information introduction
1	Regular system (only heat pump + PVs)
2	With a battery energy storge system (BESS)
3	With a thermal energy storge system (TESS)
4	With an electrothermal hybrid energy storage system (HESS)

## Model and research method

**Figure 5:**
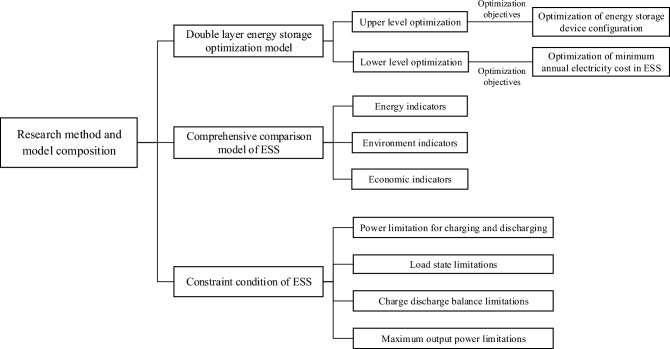
Structure of the research method and model compositions.

This study involved two main research models, namely, the double-layer optimization model and the comprehensive comparison model. The double-layer optimization model is used to achieve dual optimization of the energy storage device configuration and system energy management. The comprehensive comparison model is used to comprehensively compare and evaluate ESSs in different scenarios. At the same time, it is necessary to meet certain constraints when using ESS energy consumption simulation models. The research method and model compositions are shown in Fig. 5.

### Double-layer optimization model

In this study, we present an optimization model for a home energy system with an energy container that takes into account the total operating costs of the system. This model considers system costs holistically, improving system financial performance while ensuring safe system operation and optimizing the energy storage and management systems.

**Figure 6:**
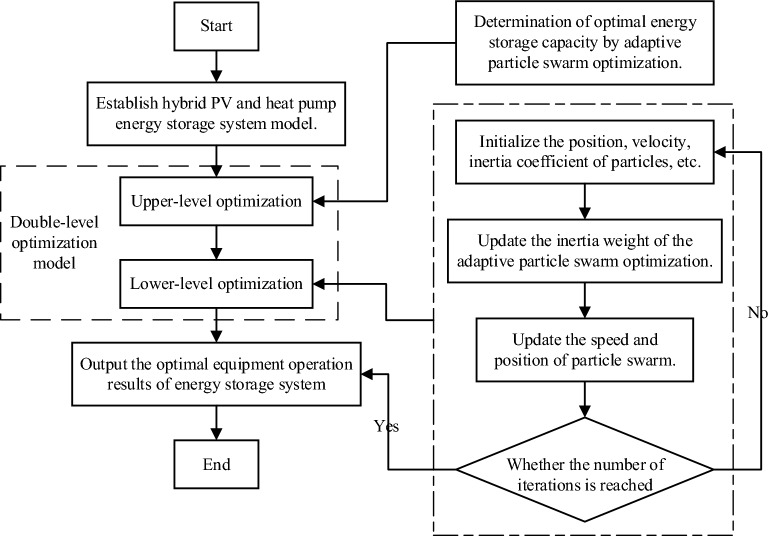
The framework of the double-layer optimization model.

The ESS optimized model has two layers, as illustrated in Fig. 6. The upper-level optimal target is to reduce the storage and procurement costs of the power grid. An adaptive particle swarm optimization(APSO) mathematical model is proposed to optimize the capacity and energy of the ESS. Particle swarm optimization (PSO) is one of the best methods for solving this problem. The random velocity vector and position of the particle are evaluated by the objective function. The particle position is set as a parameter to calculate the objective function value, and the archival optimal solution ${P}_{a}$ and the global optimal solution ${P}_{g}$ are output.

The expression is as follows:

1 $${v}_{i}^{k+1}={\omega v}_{i}^{k}+{c}_{1}\mu \left({P}_{a}^{k}-{x}_{i}^{k}\right)+{c}_{2}\eta ({P}_{g}^{k}-{x}_{i}^{k})$$

2 $${x}_{i}^{k+1}={x}_{i}^{k}+{\rho v}_{i}^{k}$$

The PSO method takes advantage of some parameters maintain its velocity and learning speed. The inertial weight factor, represented by "ω", helps to maintain the speed of particles. A single acceleration coefficient represented by "${c}_{1}$", is used to ensure that each particle learns. The global acceleration factor represented by "${c}_{2}$", is used to keep all particles learning. At the same time, a random number "$\mu , \eta$" between 0 and 1 is applied. Additionally, a restraint factor "$\rho$" is used to update the particle position, and the updated particle position is generally set to 1. Nevertheless, it is possible that the PSO method will approach a local optimization relation during the iterative procedure. To resolve this issue, we propose an APSO algorithm for optimizing fitness, which takes the basic PSO method as its basis.

The optimization expression is as follows:

3 $${P}_{i}\left(t+1\right)=\left\{\begin{array}{c}{P}_{i}(t)\left|\cdot T, f({X}_{i}(t+1))\le f({X}_{i}(t))\cdot T\right.\\ {X}_{i}\left(t+1\right), f({X}_{i}(t+1))>f({X}_{i}(t))\cdot T\end{array}\right.$$

A decay constant is introduced into the APSO algorithm, which leads to individual decay and global optimization. The decay constants of all particles are identical, but their refresh rate are different. With the increase in X, the optimum value will be renewed increasingly more frequently, and then, the optimum will be achieved.

Once the top-layer optimization is completed, the lower layer is optimized, and the overall cost of the system is optimized. Through the calculation above, the optimal energy output of each piece of equipment in the system is determined. The complete cost includes three parts: the initial input, operation, and maintenance.

4 $$min\sum {C}_{total}={C}_{inv}+{C}_{run}+{C}_{main}$$

In the optimization model, the cost of installing the PV system is denoted by "${C}_{inv}^{PV}$", the cost of installing the heat pump is denoted by "${C}_{inv}^{HP}$", and the cost of installing the energy storage device is denoted by "${C}_{inv}^{ES}$". The energy storage device consists of the initial input cost of the storage battery, denoted by "${C}_{inv}^{BEES}$", and the thermal storage equipment, denoted by "${C}_{inv}^{TEES}$".

The system operating costs includes the purchasing and sale of power from and to the public network.

5 $${C}_{run}={C}_{import}^{Grid}-{C}_{feed-in}^{Grid}$$

6 $${C}_{import}^{Grid}=\sum_{m}\sum_{h}{E}_{m,h,import}^{Grid}\times {P}_{ele}^{import}$$

7 $${C}_{feed-in}^{Grid}=\sum_{m}\sum_{h}{E}_{m,h,feed-in}^{Grid}\times {P}_{ele}^{grid}$$

where ${E}_{m,h,import}^{Grid}$ is defined as the quantity of electricity that consumers purchase from the electric network, ${P}_{ele}^{import}$ is the unit electricity price, ${E}_{m,h,feed-in}^{Grid}$ is consumers' return to the common network, and ${P}_{ele}^{grid}$ is the unit cost of the sale.

This research takes into account the maintenance costs of PVs and power storage devices.

8 $${C}_{main}={C}_{main}^{PV}+{C}_{main}^{BESS}+{C}_{main}^{TEES}$$

9 $${C}_{main}^{PV}=\sum_{m=1}{K}_{m}\left|{p}_{m}(t)\right|\Delta t$$

10 $${C}_{main}^{BESS}(t)=\sum_{i=1}{K}_{i}\left|{p}_{i}(t)\right|\Delta t$$

11 $${C}_{main}^{TESS}(t)=\sum_{x=1}{K}_{x}\left|{p}_{x}(t)\right|\Delta t$$

where ${p}_{m}(t)$, ${p}_{i}(t)$ and ${p}_{x}(t)$ denote the installation's output capacity in t; ${K}_{m}$ is the PV maintenance cost factor, expressed as 0.003USD/kWh; ${K}_{i}$ is the cell's maintenance cost factor, expressed as 0.013 USD/kWh; and ${K}_{x}$ is the maintenance cost factor for the heat storage tank, expressed as 0.007 USD/kWh.

### Comprehensive comparison model

**Figure 7:**
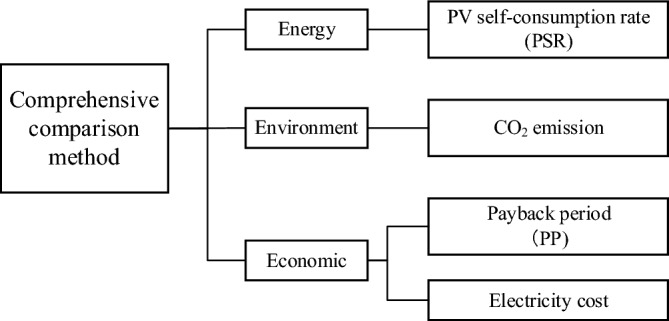
The framework of the comprehensive comparison model.

To compare and evaluate various ESSs, a comprehensive comparison model was developed to compare various aspects of system performance variation across the source cases and ESSs. Figure 7 illustrates the structure of the comprehensive comparison model, which takes into account three indicators: energy, the environment, and economics. The energy performance of the system is evaluated by the PV self-consumption rate (PSR), which can directly reflect the PV absorption capacity of the system before and after introducing the energy storage equipment. The PSR calculation expression is as follows:

12 $$PSR=\frac{{E}_{con}^{PV}}{{E}_{pro}^{PV}}\times 100\%$$

where ${E}_{con}^{PV}$ and ${E}_{pro}^{PV}$ are PV power consumption and production, respectively.

We use CO_2_ emissions to analyze the environment. Since CO_2_ tax policy implementation, CO_2_ emissions have had a direct impact on the economies of some countries. The CO_2_ emissions in this study relate only to network imports, and the calculation is as follows:

13 $${Ce}_{ele}={\sum }_{d}{\sum }_{h}\left({E}_{m,h,import}^{Grid}\times {E}_{cf}\right)$$

where ${E}_{cf}$ is defined as the CO_2_ emission coefficient of Kyushu Electric in 2018^32^.

To compare system economics, the model is evaluated by two measures, the payback period (PP) and the power price. To highlight the economic differences of the study cases in the operation process, the electricity cost in the index considers only the cost that users pay to buy electricity from the public grid to compare the economic performance from the perspective of system equipment. At the same time, to more clearly evaluate the relationship between the future income of the system and the current initial investment, the model introduces the length of the PP as the economic evaluation index of the system operation cycle:

14 $$F={CO}_{OS}-{CO}_{ESS}$$

15 $$NPV=\sum_{t=0}^{T}\frac{F-{C}_{run}}{{(1+r)}^{t}}$$

where $F$ is the yearly operating income of the ESS and ${CO}_{OS}$ and ${CO}_{ESS}$ are the yearly operating costs of the ESS under the original scheme. $r$ represents the reference rate bank return, expressed as 3.5%. $T$ represents the real lifetime of the ESS. The system net present value is represented as $NPV$; if 0, $t$ is the system PP.

### Constraint conditions

As mentioned above, the housing network model under consideration is optimized based on the ESS. In the process of modeling, consideration should be given to the status of energy in every period, such as the capacity of the device, the battery capacity, and the quantity of energy stored. Consequently, the establishment of the model must comply with the following constraints:

First, in the process of charging and discharging, it is essential that the capacity of the storage device be respected.

16 $$-{P}_{e}<{P}_{ESS}(t)<{P}_{e}$$

The energy storage device has a maximum power limit for both charging and discharging. Importantly, the cell must comply with the condition of charge restriction to avoid overcharging or discharging above safe levels.

17 $${SOC}_{min}\le {SOC}_{(t)}\le {SOC}_{max}$$

To ensure the functioning of the power storage cell, this model establishes a state of charge(SOC) value of 0.9 and 0.1. In addition, the ESS must comply with a daily charge and discharge balance to maintain flexible control over the operating status of power storage.

18 $$\int \left|{P}_{{ESS}_{d}}\right.\left(t\right)\left|{\eta }_{d}dt\right.=\int \left|{P}_{{ESS}_{c}}\right.\left(t\right)\left|{\eta }_{c}dt\right.$$

In this model, ${P}_{{ESS}_{c}}(t)$ and ${P}_{{ESS}_{d}}(t)$ are used to indicate the electric capacity of the battery. Charging and discharging efficiency is indicated by "${\eta }_{c}$" and " ${\eta }_{d}$" respectively. To avoid an excessively large variation in the power output of an energy storage device that may adversely affect the lifetime and economic performance of the device, the variation in the output power of the device is restricted as follows:

19 $$\left|{P}_{ESS}\left(t\right)-{P}_{ESS}\left(t-1\right)\right|\le \delta P$$

where ${P}_{ESS}\left(t\right)$ is the present output of the ESS, ${P}_{ESS}\left(t-1\right)$ is the most recent output power of the energy storage device, and is the maximum amount of power change per unit time.

## Results and discussion

### Comparative analysis of the energy consumption simulation results

In this study, to complement the HEMS residential energy management strategy, we introduce storage devices based on existing target home energy systems. Adding energy storage devices can improve the performance of the PVs and thermal electric pumps in the system, stabilize the system, enhance user economics, and balance grid loads.

**Figure 8:**
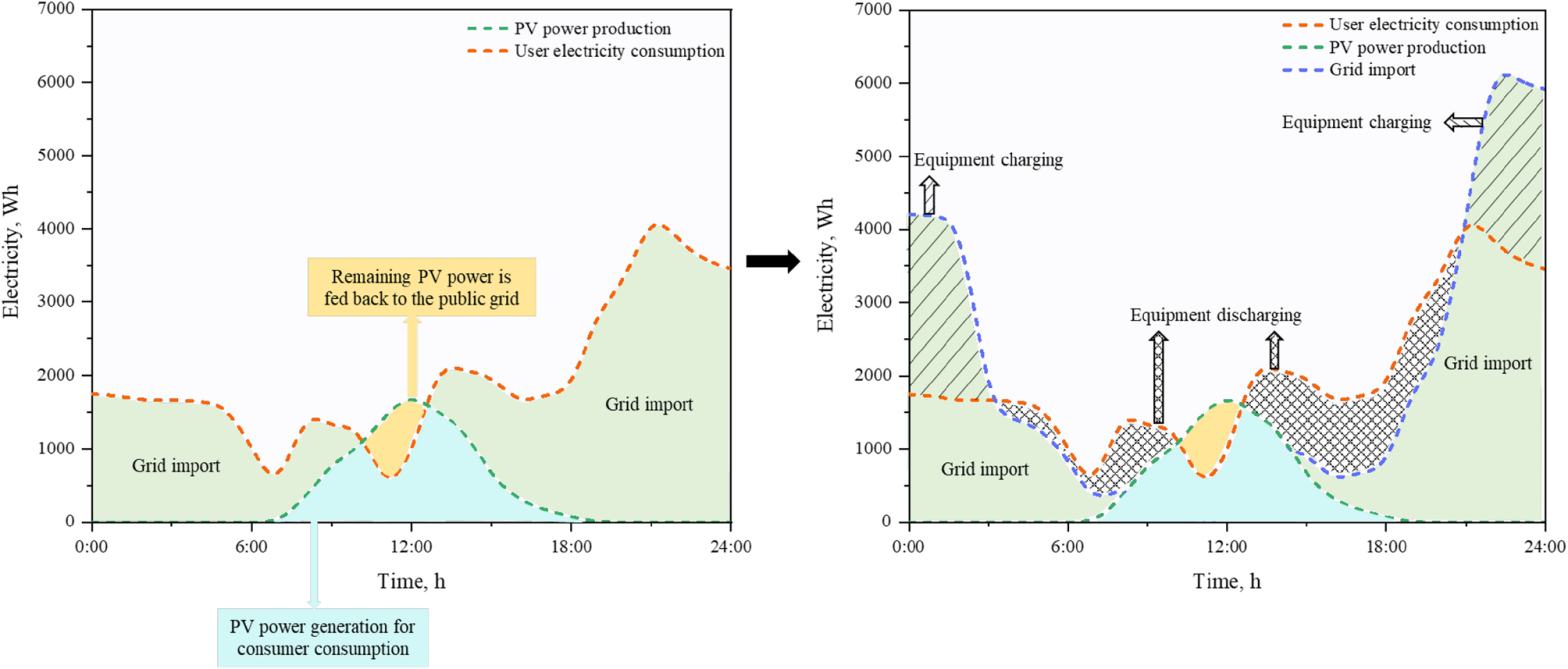
System action mechanism after introducing the energy storage device.

The TOU scheme for the target households and the special tariff data are presented in Table 3^33^. TOU is characterized by being expensive during the day but inexpensive between ten o'clock in the evening and eight o'clock in the morning. Figure 8 illustrates the simple working process of reducing electricity bills and user peaks and achieving energy accumulation by introducing devices with 24-h energy consumption changes.

**Table 3:** Specific pricing information of TOU.

Basic price (USD/contract)	Unit price (USD/kWh)		
14.85	8:00–22:00	Spring/Autumn	0.21
		Summer/Winter	0.24
	22:00–8:00		0.12

The reason for this phenomenon is that when the external power cost is high, the internal device can mainly produce power to satisfy customers' needs and return the excess power to the network. Deficits can be addressed by purchasing electricity from the public network. During periods of low electricity prices, to meet consumers’ electricity needs, savers can be recharged, and this saved electricity can be delivered to consumers when electricity prices rise in the future.

Moreover, when the device is running, the power storage device and the system can be used in coordination to maintain optimal operating conditions, thereby reducing the operating cost. The storage device should be optimized to make it usable in January, which is the month in which the largest amount of electricity is consumed. The best result that meets users' maximum energy consumption can be selected. Table 4 displays the optimal capacity of the device using a model that optimizes the storage size. On the basis of the optimized results, we performed a four-year simulation. Four days and 4 seasons were chosen as representative dates for analysis and comparison of the performance of various pieces of equipment each hour.

**Table 4:** Case introduction.

Case	1	2	3	4
BESS size(kWh)	–	8.2	–	5.6
TESS size(kWh)	–	–	4.7	3.4

#### Case 1: regular system

**Figure 9:**
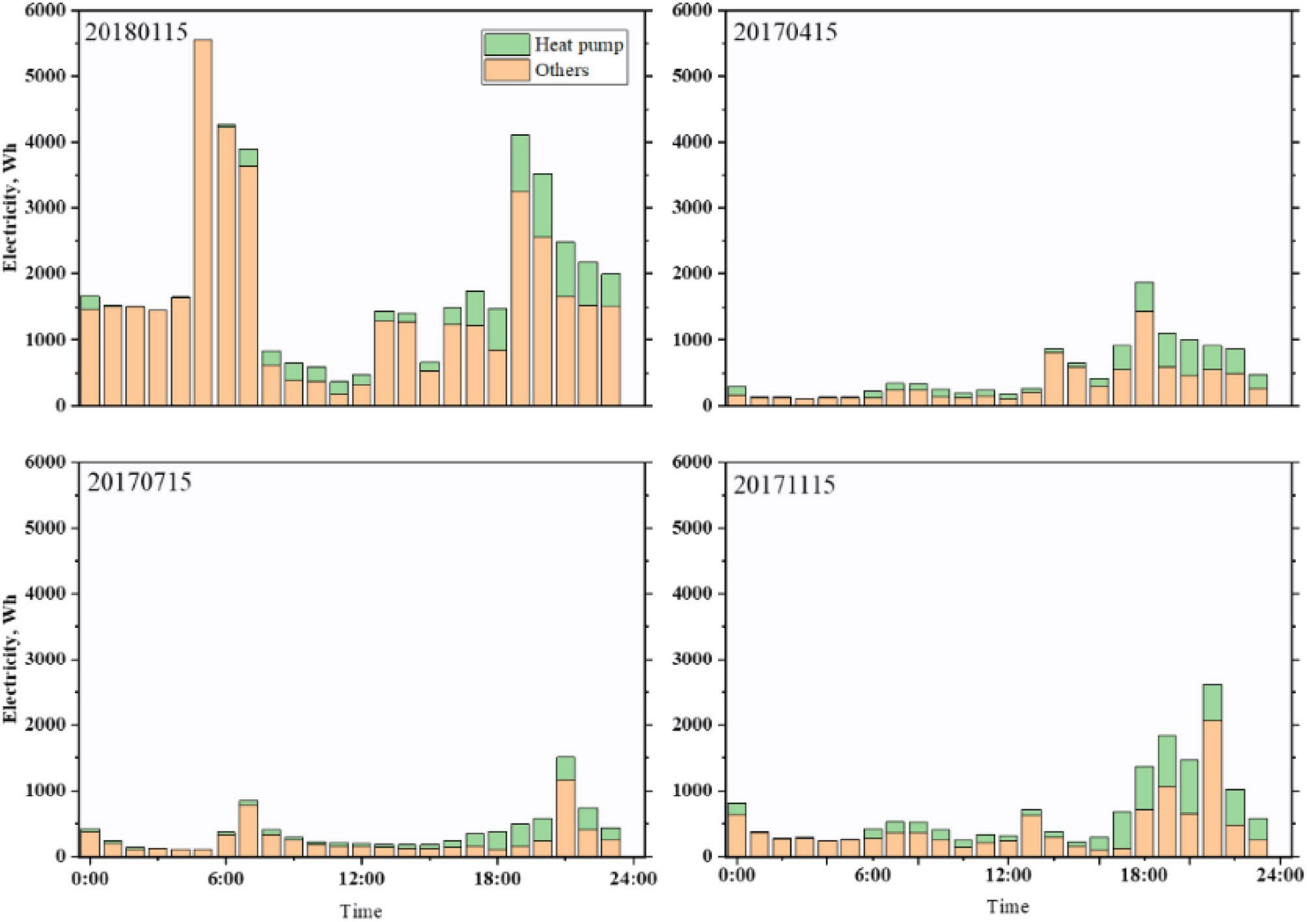
User hourly electrical load on typical day in Case 1.

The first case focuses on a present household's primary power supply, where PVs and thermal pumps are utilized to satisfy customers' electrical and thermal requirements. Figure 9 displays users' hourly energy load over a typical four-day period. In general, the maximum amount of power and heat use occurs in the evening, but in the winter, it is much greater than that in other seasons. Changes in user load appear to be influenced by the time of year and users' energy consumption habits. Heat pumps also consume more energy at night, but they generate electricity during the day.

#### Case 2: with a BESS

**Figure 10:**
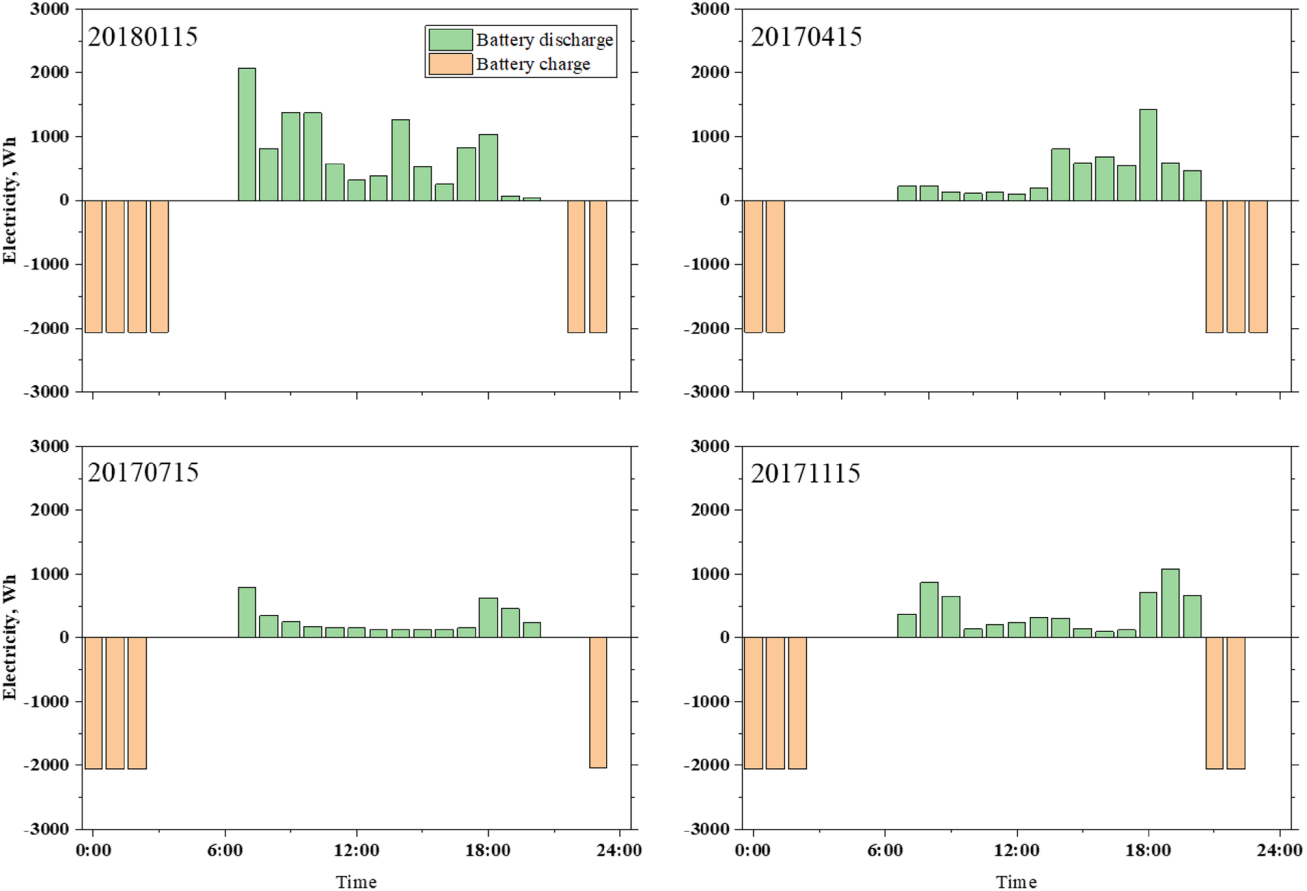
Battery charge and discharge on a typical day in Case 2.

For the purpose of this research, we designed an ESS for Case 2. On the basis of the optimized results, it was decided that the optimum cell dimensions are 8.2 kWh. Figure 10 shows the average number of hours of charging and discharging. Considering these constraints, the charging and discharging modes of the battery cannot be simultaneous. Due to the choice of the TOU electricity rate, the price at night is relatively low, and consumer needs are reduced by 10 p.m. Thus, the charging times are set at low cost times, and the electricity needs of customers are satisfied at night. Daytime is reserved for generating electricity when solar power is insufficient and for reducing the amount of power the system receives during periods of high electricity prices. Battery charging and discharging are mainly determined by typical hourly energy consumption, but seasonality also plays a significant role. Additionally, PV power generation has an impact on the charge and discharge capacity of the battery. Due to the abundant solar energy in the summer, the battery discharge is much lower during the day than in winter when electricity supply is limited.

#### Case 3: with a TESS

**Figure 11:**
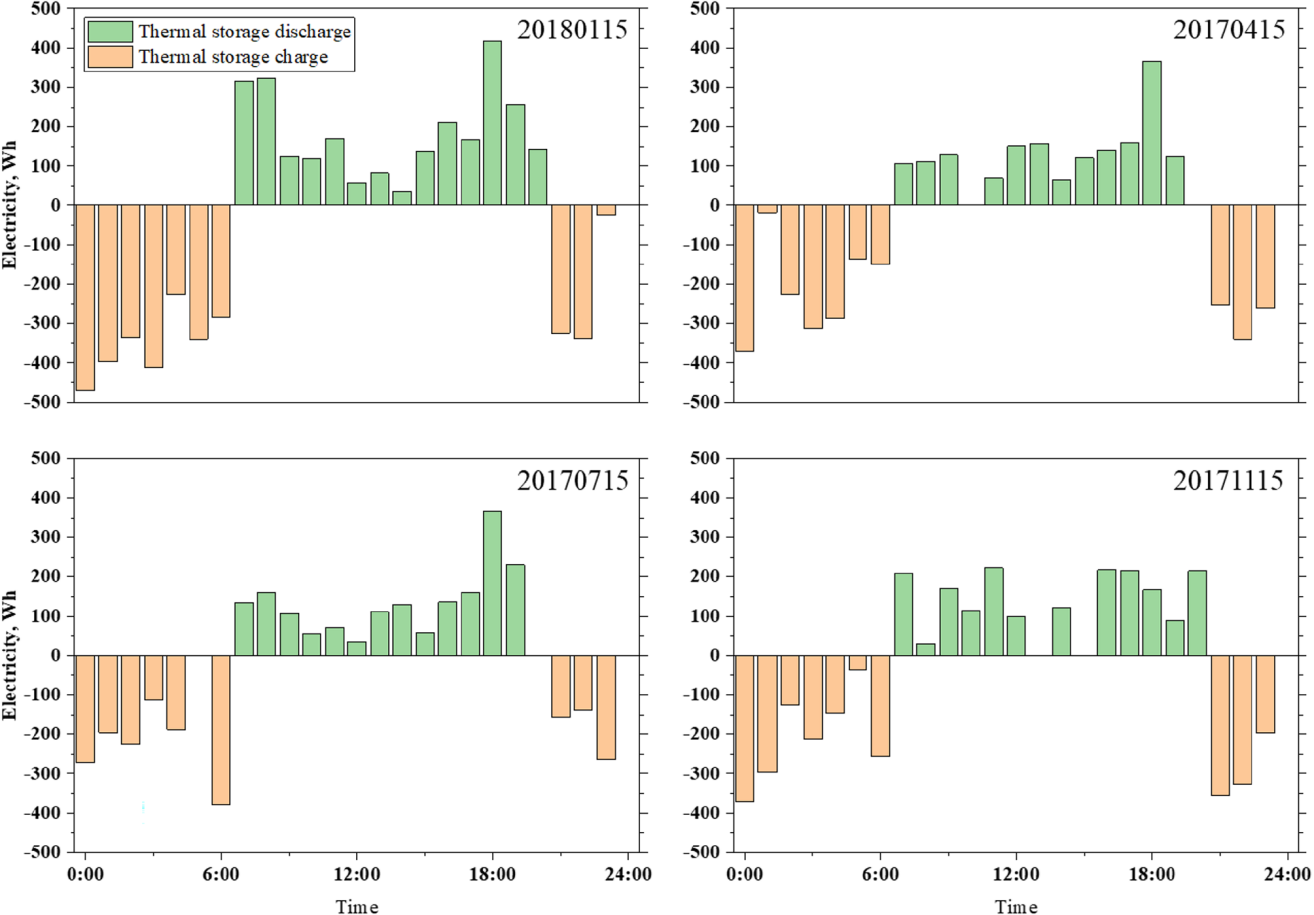
Thermal tank charge and discharge on a typical day in Case 3.

In the initial research, the home was heated by a heat pump. As shown in Fig. 9, to meet users' needs, the heat pump must operate for most of the day, resulting in energy consumption. Peak power consumption occurs from 6:00 to 10:00 in the evening, when energy consumption is higher during peak price periods, Case 3 is designed as a TESS with an optimized thermal battery capacity of 4.7 kWh. Figure 11 illustrates a typical hourly battery charging and discharging. The battery works in conjunction with the heat pump, and hot water from the heat pump is used to cover low-cost heat needs, with some stored in the battery. To reduce the electricity purchased by consumers for high-cost hot water needs, the thermal battery releases electricity during high-price periods, providing hot water to consumers. Compared with other seasons, there is no significant difference in the charging or discharging capacity in winter. Due to the introduction of thermal batteries, heat pumps are more active at night than during the day, and the purchased power increases within a low price range. A container is used to store heat, and users understand the purpose of reducing peaks, filling gaps, and reducing costs.

#### Case 4: with a HESS

**Figure 12:**
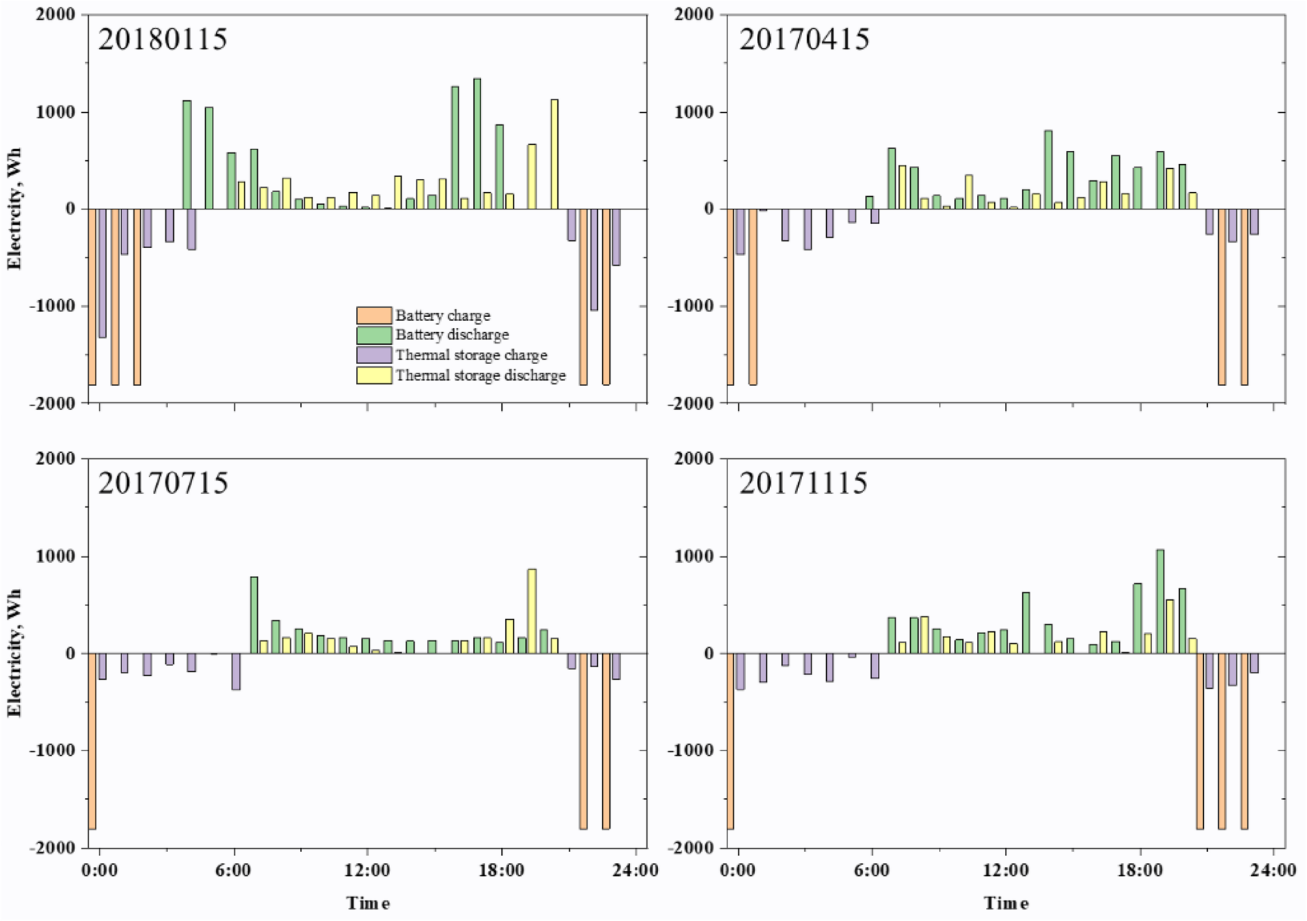
Energy storges equipment charge and discharge on a typical day in Case 4.

On the basis of the analysis above, an energy storage unit can be added in conjunction with other devices to control the maximum energy consumption of customers and to reduce the purchase power when the cost is high. However, the performance of storage devices varies, and when introducing batteries and thermal storage tanks into the home grid, the power of the devices can differ. In Case 4, the HESS combines batteries and thermal storage tanks. Figure 12 illustrates a typical day's charging and discharging hours. Compared with Case 2 and Case 3, the hybrid power generation system has more flexibility in charging and discharging. In the HESS, the difference between day and night is predominant. However, when the heat storage battery device is introduced, the energy output rate of the battery is higher than that in Case 2. The reason is that when the energy storage device apparatus operates together with other devices, there is an interaction between the cell and the thermal reservoir. Moreover, there is a problem in which the cell sends electricity to the hot-water pump, thus, it cannot meet the needs of customers during the day.

### Comparative analysis of energy storage system performance

A comparison of a typical day's performance reveals only the difference between the number of hours during which electricity is consumed and the quantity of power generated. To understand the effects of different devices and ESSs, we use an optimized model to simulate the yearly energy output and consumption. The simulation results are analyzed and compared from three perspectives: energy, the environment, and economics.

**Figure 13:**
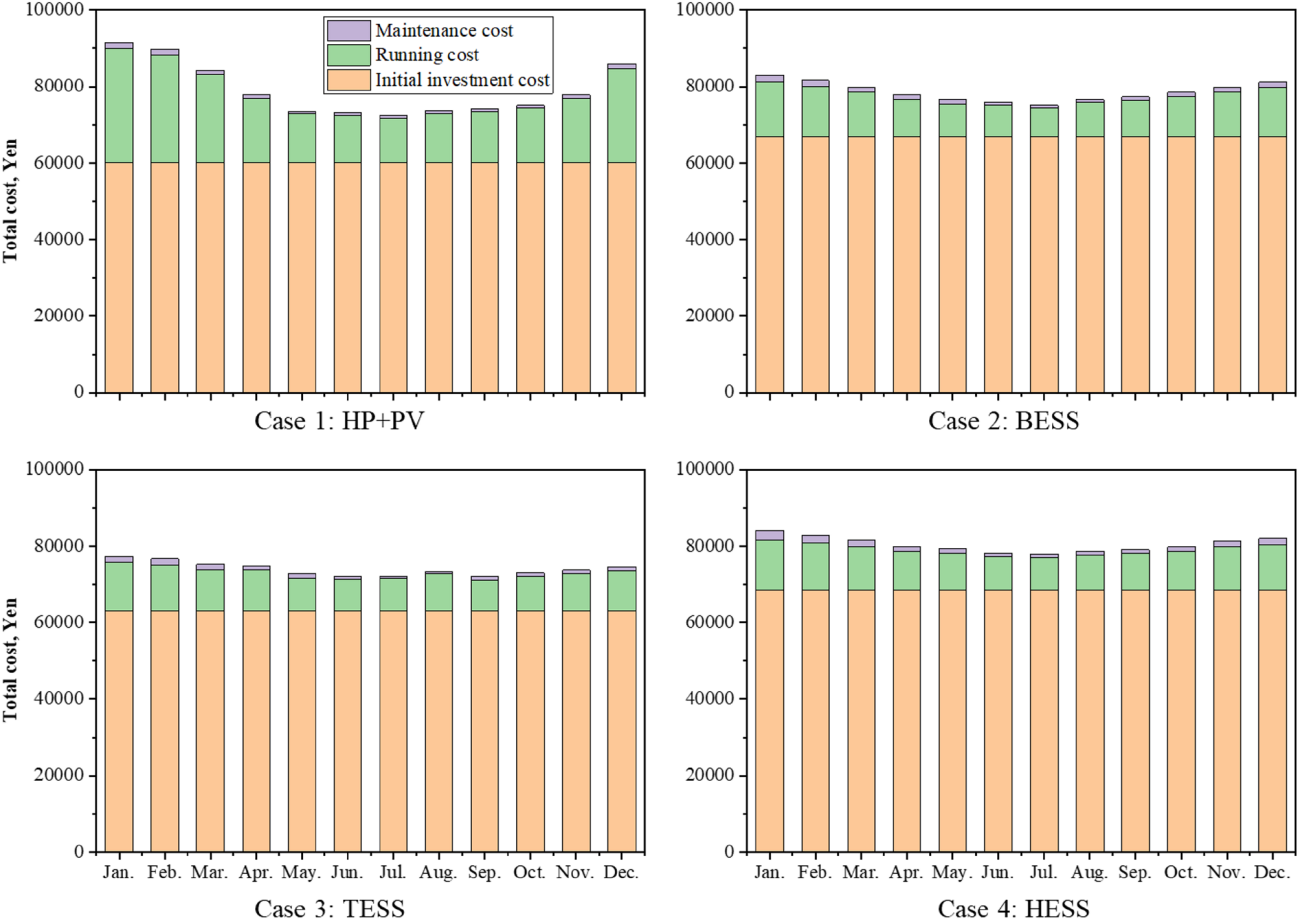
Monthly comparison of the total annual cost.

**Figure 14:**
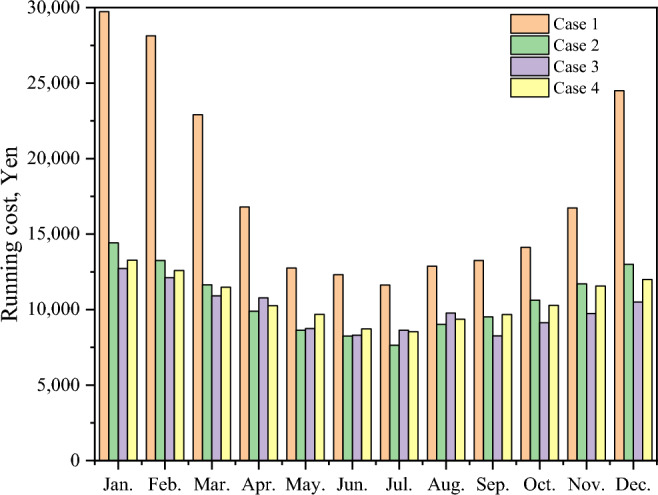
Monthly comparison of the running cost.

**Figure 15:**
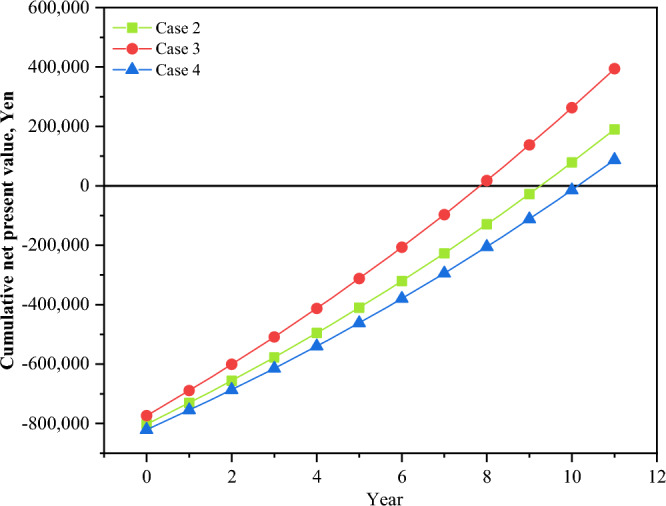
Payback year comparison of Cases 2, 3 and 4.

Figure 13 shows a comparison of the total costs of the four cases each month, including investment, operation, and maintenance costs. Because it is expensive to buy equipment, the proportion of the investment cost is far greater than that of the others. In today's market, PV components and batteries are more expensive. To achieve a stable energy supply, lower costs, promote energy allocation and create a new society, a series of subsidy policies has been introduced for PVs, batteries and HEMS homes to reduce CO_2_ emissions from renewable sources and combat global warming^34^. On July 1, 2012, Japan launched a fixed grid electricity price policy (FiT) to encourage people to earn profits by selling the remaining electricity generated by PVs back to the grid at higher prices, which effectively promotes the development of PV systems. In the Japanese residential market, 2.2 million households currently own rooftop PVs. Due to the combination of subsidy policies, the investment return period for installing rooftop PVs is approximately within 10 years, which is similar to the FiT subsidy period. Therefore, the investment return rate for installing residential PVs is widely accepted by people. After calculating the total cost of the four cases based on the subsidy policy, the results show that the price of subsidized batteries is still relatively high. Investment costs differ to some extent when energy storage equipment is introduced; however, in three cases the growth is lower, and in four cases, the growth is greater. In addition to the fixed increase in the cost of investment, there is also a certain reduction in the cost of using energy batteries. Figure 14 shows a comparison between the four current spending levels each month. ESSs are economically advantageous in terms of running costs, particularly in winter. However, the introduction of a TESS may increase winter maintenance costs. Conversely, in June, July, and August, when temperatures gradually rise, the operating costs of BESSs become more economically favorable. A HESS has a relatively balanced feature set that reduces operating costs. The results also show that there are more winters than summers, but the seasonal factors are significantly greater than in Cases 2 and 3. The operating costs of Cases 2, 3, and 4 are 36.92%, 44.68%, and 34.76%, respectively. Figure 15 displays a comparison of PPs and ESSs. Case 3 has the shortest PP, 7.84 years, while Case 4 has the longest PP, 10.29 years.

**Figure 16:**
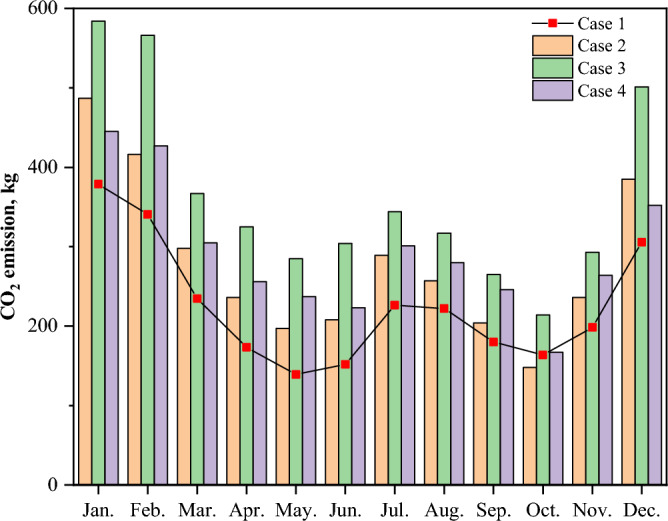
Monthly comparison of CO_2_ emissions.

Figure 16 illustrates the results of the four cases of CO_2_ emissions to compare environmental indicators. Since ESSs can store electricity at low-cost electricity prices, the CO_2_ emissions differ among the four systems due to the differences in the number of storage devices compared to the original system. Case 4 consumes a large amount of electricity in winter, which is beneficial for the environment, while Case 2 produces less CO_2_ in winter. Concurrently, the CO_2_ emissions in Case 3 are apparently higher than in Cases 2 and 4. Table 5 presents the comparison results of the energy, environmental, and economic indicators. From an economic perspective, Case 3 is the most favorable as it takes 7.84 years to pay for itself. From an environmental standpoint, comparing the annual CO_2_ emissions of the four cases, we see that those of Case 2 are the lowest. However, more energy storage could increase the capacity of the solar system to absorb solar energy. On the other hand, Case 4 has a PSR of 54.95% annually.

**Table 5:** Specific pricing information of TOU.

	Case 1	Case 2	Case 3	Case 4
PSR	14.31%	38.14%	33.46%	54.95%
CER (kg)	2714	3361	4455	3503
PP (year)	–	9.32	7.84	10.29
Total electricity cost (USD)	8836.93	8493.24	8146.89	8683.35

### Sensitivity analysis

#### Sensitivity analysis of the electricity price

As a highly efficient device for generating electricity using renewable energy, PV power generation equipment is relatively expensive to start with. To offset these high costs, and to encourage a mixture of renewables and distribution, the government has created several subsidies, including the FiT policy^35^. Because of the dual development of policy and technology, the number of domestic solar power stations has greatly increased, and these stations have played an active role in environmental improvement and economic growth. After a decade of Energy Tax Directive (ETD) application, however, the green rate tends to fall as solar generation grows^36^.

**Figure 17:**
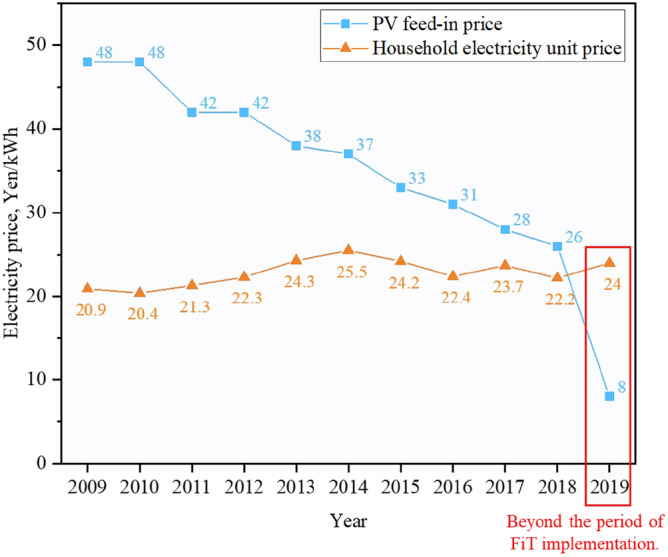
Price fluctuations in selling and purchasing electricity from the public grid.

**Figure 18:**
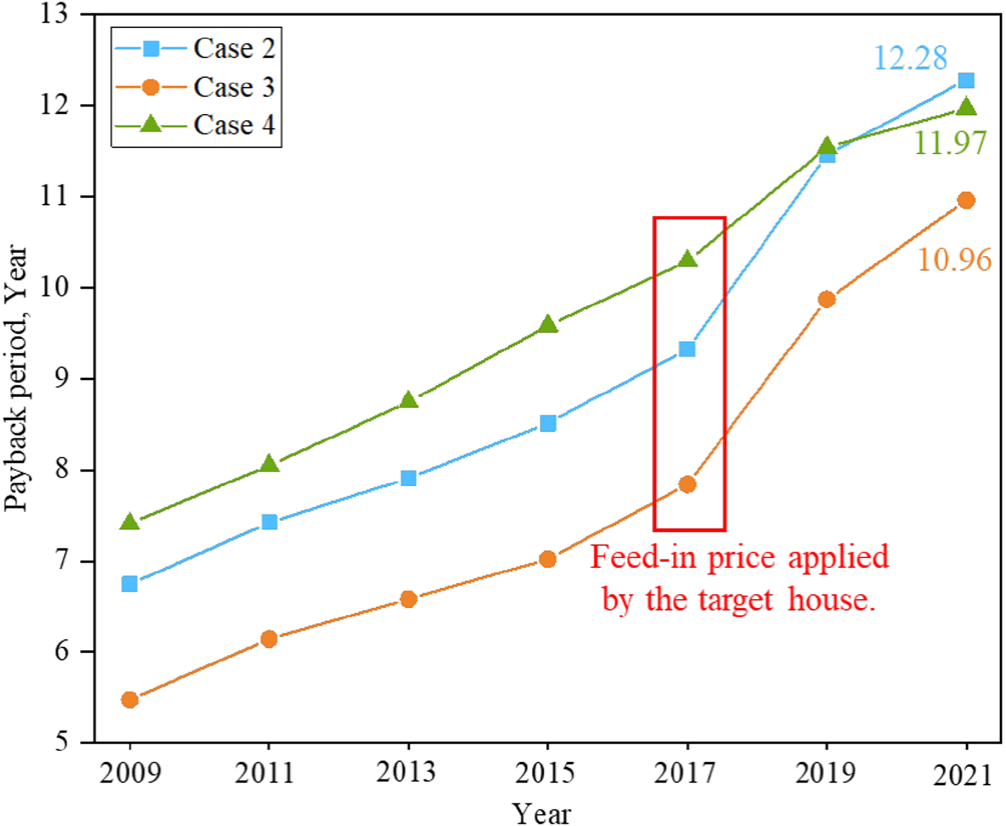
Comparison of system payback years combined with feed-in price fluctuations.

Figure 17 displays the price fluctuations in electricity supply from the power grid during policy implementation. Since the implementation of the FiT policy by the Japanese government in 2009, the unit price of PV feed-in price has been decreasing year by year. Especially in 2019, there was a significant price reduction. This has also led to fluctuations in the unit price of electricity used by residents. Solar energy is the main source of energy for homes, and some of its economic advantages are reflected in preferential tariffs. Based on this phenomenon, we calculated the PP of ESSs from 2009 to 2021, and the comparison results are shown in Fig. 18. The results indicate that the decrease in PV feed-in price has extended the PP of ESSs, and the recovery years of all three systems have shown varying degrees of increase. Due to the lower initial investment in the system, the number of years of recovery in Case 2 has always been less than in Cases 3 and 4. But compared to Case 2 and Case 3, Case 4 has the slowest growth rate. Therefore, although Case 4 had more system recovery cycles in the previous years than Case 2 and Case 3 due to higher initial investment, with the continuous decline in PV feed-in price, Case 4 has begun to show economic advantages, and the number of system recovery years in 2021 has been less than Case 3.

#### Sensitivity analysis of the carbon tax

**Figure 19:**
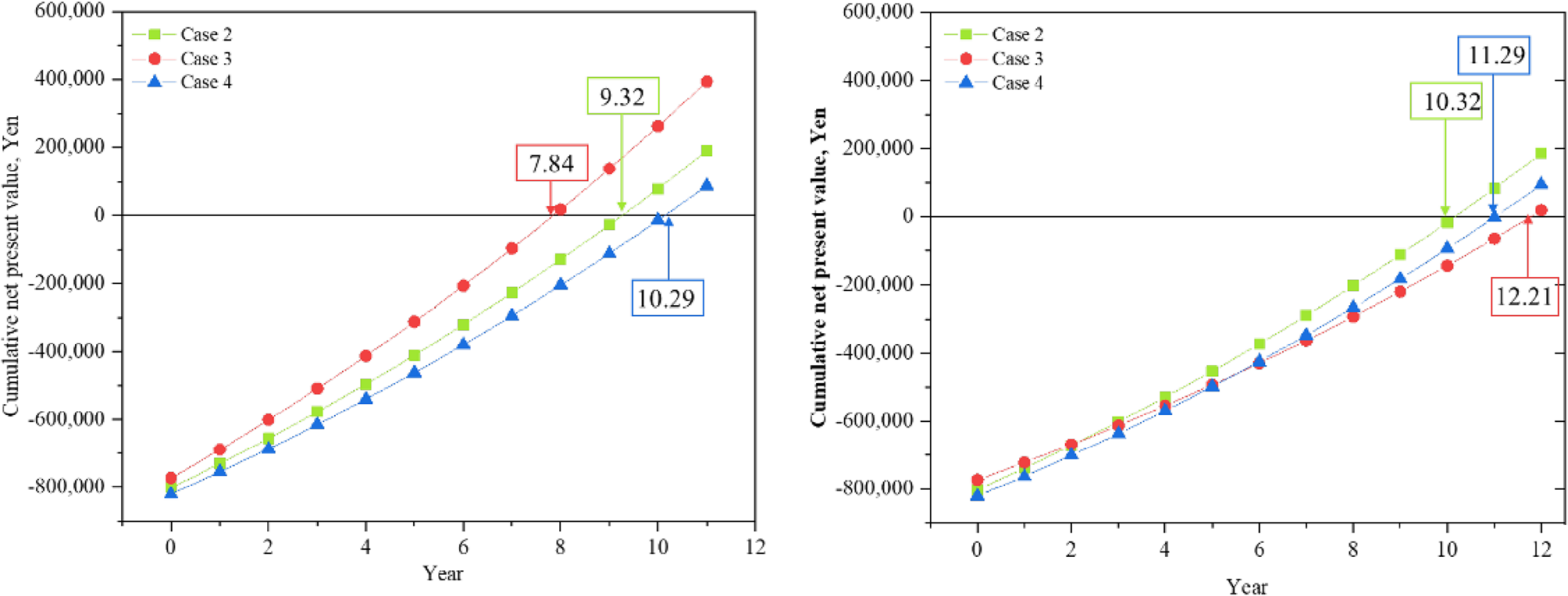
PP comparison before (left) and after (right) the carbon tax.

In 1973, Wilson proposed the concept of a CO_2_ tax to address the greenhouse effect of CO_2_ emissions^37^. By 2020, 31 countries had implemented or planned to implement carbon taxes, while more than 100 cities have committed to becoming carbon neutral by 2050^38^. Starting in 2012, Japan introduced a CO_2_ tax on certain firms, with the intention of introducing full CO_2_ taxation by 2022 as an effective tool for achieving a low-carbon society^39^. When Japan first implemented the carbon tax, to prevent a sharp rise in short-term pressure, the government raised the tax rate to create an adjustment period for citizens. As of 2020, the up-to-date carbon tax price in Japan was 0.039 USD/kg CO_2_. Taking into account actual cost of carbon taxation in Japan, we evaluated the PPs of ESSs. Figure 19 shows the PP scheme compared with CO_2_ taxation. By comparing the calculation results of recycling years, it can be seen that the recycling years of the three ESSs have all increased to varying degrees after the introduction of carbon tax. However, based on the growth trend of recycling years, compared to Case 3 and Case 4, Case 2 presents a better environmental advantage. After the introduction of carbon tax, it only takes 10.32 years to recover costs. On the contrary, Case 3 has the longest recycling year, which is 12.21 years.

## Conclusions

As energy storage technologies become more prevalent in home energy systems, collaboration between devices and users creates more opportunities to optimize the system. The complex coupling relationship between different energy storage devices and their energy consumption characteristics also causes composite energy storage to have greater optimization and development potential than single-type energy storage devices. In this study, an independ-ent house with PVs and a heat pump in the Jono Zero Carbon Advanced Urban Area is selected as the research object. Based on the APSO algorithm, a double-level optimization model is proposed for the capacity of energy storage equipment and the annual energy consumption of the system. This model is intended to reduce peak pressures while lowering energy costs and optimizing system efficiency, thereby increasing the operational reliability of the system. At the same time, a composite energy storage comprehensive comparison model is established, and four cases with different energy storage equipment are designed to compare and evaluate the model from three perspectives: energy, the environment and economics. On the basis of our present analysis, the following conclusions can be drawn:

1. In the scenario of applying different energy storage equipment, the equipment capacity is optimized, and the optimal size is obtained through the upper-layer optimization model. Then, the annual equipment output of the system is simulated based on the optimal size. The hourly energy consumption simulation results reveal that the addition of energy storage equipment plays a positive role in reducing users' peak load and electricity purchase cost and can cooperate with PVs and heat pumps.
2. The four cases show different characteristics of energy storage equipment. The charging and discharging of BESSs are greatly affected by PV power generation, which reflects obvious seasonality. The charging of the TESS in winter is much higher than that in other seasons, and after introduction, the operation time of the heat pump changes from all-day to concentrated work at night. Compared with a single ESS, the charging and discharging of a HESS is more flexible, and the difference between daytime and nighttime electricity prices is more pronounced.
3. The comprehensive comparison results show that the TESS has economic advantages, with a system PP of 7.84. Regarding environmental performance, the addition of energy storage equipment leads to an increase in system carbon emissions to varying degrees, among which the increase of the BESS is the smallest.
4. In terms of energy performance, the HESS has the highest PSR and can consume more PV power generation than the BESS and TESS. According to the sensitivity analysis results of electricity price fluctuations and the carbon tax, the PPs of ESSs increase to varying degrees under the background of gradually decreasing feed-in prices. However, the growth rate of the HESS is the smallest, and it will reflect greater economic advantages in the future as the feed-in price continues to decline. Meanwhile, the possibility of introducing a carbon tax in the future highlights the environmental advantages of the BESS and HESS.

At present, although the HESS cannot recover costs within 10 years, based on price fluctuations and the gradual introduction of the carbon tax, the HESS shows development potential in terms of both economics and the environment. A higher PV absorption capacity also provides greater optimization space for further combinations of HESSs and PVs. However, based on the current analysis results, the high price of PVs and batteries is one of the main reasons for the extension of the PP of the system. To promote the collaborative application of energy storage equipment and renewable energy on the user side, the government has successively implemented subsidy and incentive policies. It is hoped that there is further cooperation with energy storage equipment according to corresponding policies, that the role of user-side energy storage in demand management and grid peak shaving is leveraged, and that the dual optimization of the economy and the environment is realized.

## Data Availability

The data that support the findings of this study are available from the corresponding author, [Xiaoyu Ying, zhaoxy@zucc.edu.cn], upon reasonable request.

## Abbreviations

ESS: Energy storage system
PP: Payback period
BESS: Battery energy storage system
${P}_{i}$: Historical optimal solution
TESS: Thermal energy storage system
${P}_{g}$: Global optimal solution
HESS: Electrothermal hybrid energy storage system
$\omega$: Inertia weight coefficient
PV: Photovoltaic
${c}_{1}$: Individual acceleration coefficient
TOU: Time-of-use
${c}_{2}$: Global acceleration factor
EV: Electric vehicle
$$\mu , \eta$$: Random number
HEMS: Home energy management system
$$\rho$$: Constraint factor
METI: Ministry of Economy, Trade and Industry
$$T$$: Attenuation constant
FiT: Feed-in-tariff
$${C}_{total}$$: Total cost
APSO: Adaptive particle swarm optimization
${C}_{inv}$: Installation investment cost
PSO: Particle swarm optimization
$${C}_{run}$$: Running cost
PSR: PV self-consumption rate
$${C}_{main}$$: Maintenance cost
